# Impact of the numbers of injections of platelet rich plasma on the clinical outcomes in patients with knee osteoarthritis: A protocol for an updated network meta-analysis

**DOI:** 10.1097/MD.0000000000024250

**Published:** 2021-01-08

**Authors:** Pan Wang, Kai Li, Zuxin Jiang, Beiming Qiu, Cheng Nie, Hongsheng Luo, Zhengjiang Li

**Affiliations:** a Department of Orthopedics, the Second People's Hospital of Yibin, Yibin; b Department of Orthopedics, Chengdu Fifth People's Hospital, Chengdu, China.

**Keywords:** intra-articular injection, knee osteoarthritis, network meta-analysis, platelet-rich plasma, protocol

## Abstract

**Background:**

Two published meta-analyses have investigated the effects of the number of injections of platelet rich plasma (PRP) on clinical outcomes in knee osteoarthritis patients, however conflicting findings were generated.

**Methods:**

We will systematically search PubMed, Embase, and China National Knowledgement Infrastructure (CNKI) to capture additional eligible studies. After screening citations, extracting essential data, assessing the risk of bias, we will use RevMan software and Open BUGS to perform head-to-head and network meta-analysis of pain alleviation and improvement of joint functionality, respectively.

**Discussion:**

Knee joint osteoarthritis (KOA) is the main cause of joint degeneration in elderly, which seriously reduces patients quality of life. Although intra-articular PRP has been extensively prescribed to treat KOA, a definitive conclusion about the appropriate number of injections has not yet been generated in published meta-analyses. The present updated network meta-analysis will comprehensively answer this question.

**Ethics and dissemination:**

We will communicate our findings through participating in academic conferences or submiting it to be considered for publication in peer reviewed scholar journal.

**INPLASY registration number:**

We registered this protocol in International Plateform of Registered Systematic Review and Meta-Analysis Protocols (INPLASY) platform and obtained an identifier of INPLASY2020110043 (https://inplasy.com/inplasy-2020-11-0043/).

## 1 Introduction

Knee osteoarthritis (KOA) is a degenerative disease,^[1]^ which was associated with cartilage degeneration and exfoliation, and subchondral bone hyperplasia, knee pain, joint instability and functional limitations.^[2–4]^ As a common disabling disease, KOA has been estimated to affect more than 250 million people worldwide.^[5]^ It is noted that articular cartilage regeneration is very difficult; once damaged, it is difficult to repair.^[6]^ And thus, KOA can seriously negatively impact the patients quality of life.^[7]^ Although knee replacement surgery provides an effective solution for severe knee KOA,^[8]^ the Osteoarthritis Society International (OARSI) recommends conservative treatment rather than surgery as the first-line management solution for KOA, which emphasizes the importance of conservative treatment in the treatment of KOA.^[9]^

As one of the conservative treatments, injectable medications such as hyaluronic acid (HA), steroids, nonsteroidal antiinflammatory drugs, and platelet-rich plasma (PRP) have been proved having ability of promoting the regeneration in tissue and alleviation of symptoms.^[10–12]^ Among injectable options for symptom relief and functional improvement in patients with knee OA, PRP has increased in popularity in recent years.^[13]^ Several systematic reviews and meta-analyses have further determined the potential of PRP in treating KOA.^[1,2,14–22]^

After demonstrating the efficacy of PRP for the treatment of KOA, researchers and practitioners changed attention to further explore the optimal numbers of PRP injection. To date, several randomized controlled trials^[23–29]^ focused on this topic have been reported, and a recent meta-analysis^[30]^ has also compared clinical effectiveness of single versus multiple injections of platelet-rich plasma in the treatment of knee osteoarthritis and suggested that a single injection was as effective as multiple PRP injections in pain improvement; however, multiple injections seemed more effective in joint functionality than a single injection at 6 months. Moreover, a recent network meta-analysis also investigated the comparative efficacy of single between multiple injections for the treatment of KOA, and found no significant difference of these two regimes for pain relief and improvement of joint function.^[1]^

Unfortunately, 2 previous meta-analyses all missed some eligible studies,^[23,28]^ which obviously impaired the robustness of pooled results. Moreover, these 2 meta-analyses^[1,30]^ only investigated the comparative efficacy of single and multiple injections, multiple injections including double and triple options were not investigated separately. Thus, previous meta-analyses have limited reference value for making decision in clinical practice. Considering these issues, we designed this updated study with traditional meta-analysis and network meta-analysis techniques to further investigate the role of multiple injections and comparative efficacy of double and triple injections among KOA patients.

## 2 Methods

We registered this protocol on the International Plateform of Registered Systematic Review and Meta-Analysis Protocols (INPLASY) website and received a register number of INPLASY2020110043 (https://inplasy.com/inplasy-2020-11-0043/), and thus our protocol has been funded through a protocol registry. We will use the recommendations in the Cochrane handbook^[31]^ as the methodological framework to instruct performing this updated network meta-analysis. After finishing all statistical analyses, we will also use the preferred reporting items for systematic review and meta-analysis protocols (PRISMA-P) 2015 statement to guide reporting all results.^[32]^ We will perform all analyses based on previous studies, and thus no ethical approval from the institutional review board and patients informed consent will be required.

### 2.1 Identification of studies

**Figure 1:**
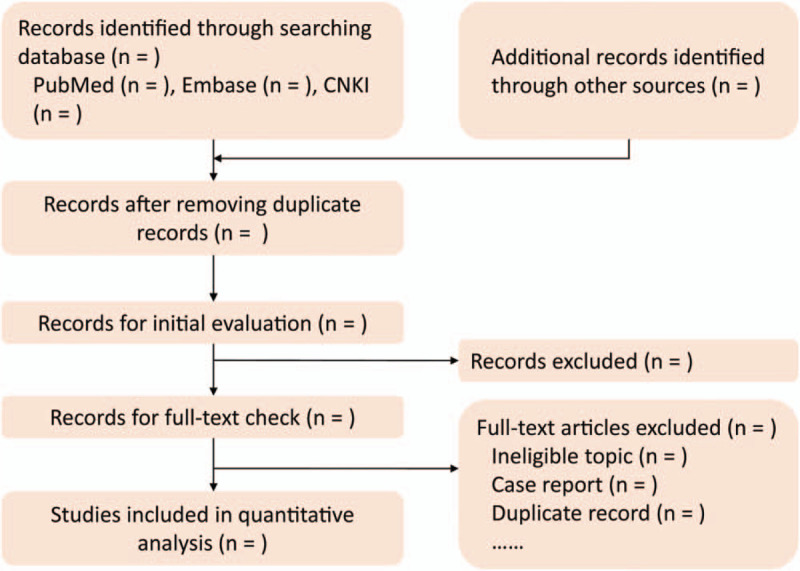
Flow diagram of study retrieval and selection. CNKI, China National Knowledgement Infrastructure. Additional records will be identified from previous meta-analysis.

Because previous topic-related meta-analysis searched all potential citations published between 1970 to July 2019,^[30]^ therefore we will perform an additional search to capture any potential records reported between August 2019 and October 2020. The additional search will be conducted by 2 independent reviewers in PubMed, Embase, and China National Knowledgement Infrastructure (CNKI). We will use the combination of text words and MeSH terms to construct a search strategy and amend to be applicable to individual database according to the unique requirements of each database. The search strings of PubMed and Embase were documented in Table 1. We will also manually check the reference lists of all eligible studies and meta-analyses tin order to include any potential studies. The process of searching and selecting potentially eligible studies will be delineated in Figure 1. Any disagreements about identification of studies will be resolved through consulting a third senior reviewer.

**Table 1:** Search strategy.

1. Search strategy of PubMed	
Search number	Search terms
10	((((“Osteoarthritis, Knee” [Mesh]) OR ((((Knee Osteoarthritides [Title/Abstract]) OR (Knee Osteoarthritis [Title/Abstract])) OR (Osteoarthritis of Knee [Title/Abstract])) OR (Osteoarthritis of the Knee [Title/Abstract]))) AND ((“Platelet-Rich Plasma”[Mesh]) OR ((Platelet-Rich Plasma [Title/Abstract]) OR (Platelet Rich Plasma [Title/Abstract])))) AND (random^∗^)) AND ((“2019/08/01” [Date - Entry]: “3000” [Date - Entry]))
9	(“2019/08/01” [Date - Entry]: “3000” [Date - Entry])
8	(((“Osteoarthritis, Knee” [Mesh]) OR ((((Knee Osteoarthritides [Title/Abstract]) OR (Knee Osteoarthritis [Title/Abstract])) OR (Osteoarthritis of Knee [Title/Abstract])) OR (Osteoarthritis of the Knee [Title/Abstract]))) AND ((“Platelet-Rich Plasma” [Mesh]) OR ((Platelet-Rich Plasma [Title/Abstract]) OR (Platelet Rich Plasma [Title/Abstract])))) AND (random^∗^)
7	random^∗^
6	(“Platelet-Rich Plasma” [Mesh]) OR ((Platelet-Rich Plasma [Title/Abstract]) OR (Platelet Rich Plasma [Title/Abstract]))
5	(Platelet-Rich Plasma [Title/Abstract]) OR (Platelet Rich Plasma [Title/Abstract])
4	“Platelet-Rich Plasma”[Mesh]
3	(“Osteoarthritis, Knee” [Mesh]) OR ((((Knee Osteoarthritides [Title/Abstract]) OR (Knee Osteoarthritis [Title/Abstract])) OR (Osteoarthritis of Knee [Title/Abstract])) OR (Osteoarthritis of the Knee [Title/Abstract]))
2	(((Knee Osteoarthritides [Title/Abstract]) OR (Knee Osteoarthritis [Title/Abstract])) OR (Osteoarthritis of Knee [Title/Abstract])) OR (Osteoarthritis of the Knee [Title/Abstract])
1	“Osteoarthritis, Knee” [Mesh]

2. Search strategy of Embase	
Search number	Search terms
#9	#8 AND (2019:py OR 2020:py)
#8	#3 AND #6 AND #7
#7	random^∗^
#6	#4 OR #5
#5	“thrombocyte rich plasma”/exp OR “platelet-rich plasma cell”/exp
#4	“platelet rich plasma”: ti,ab,kw OR “thrombocyte rich plasma”: ti,ab,kw OR “platelet-rich plasma”: ti,ab,kw
#3	#1 OR #2
#2	“knee osteoarthritis”/exp
#1	“knee osteoarthritides”: ti,ab,kw OR “knee osteoarthritis”: ti,ab,kw OR “osteoarthritis of knee”: ti,ab,kw OR “osteoarthritis of the knee”: ti,ab,kw

### 2.2 Selection criteria

We will develop the inclusion and exclusion criteria according to the previous meta-analysis.^[30]^ Generally speaking, patients who were clinically and radiographically diagnosed with KOA defined by any recognized diagnosis criteria, and with a minimum follow-up of 3 months were deemed to be eligible. We will only consider randomized controlled trials (RCTs) investigating the effect of the number of PRP injections regardless of category on clinical outcomes including pain (measured with visual analog scale [VAS] or visual numerical scale [VNS]) and joint function (measured with Western Ontario and McMaster Universities Arthritis Index [WOMAC], International Knee Documentation Committee [IKDC] subjective knee evaluation form, or Lequesne index [LI]) among KOA patients. Only research reported in English or Chinese language will be considered to be eligible.

We will exclud review, case report, case series, and observational studies including quasi-experimental research, cohort study, case-control study, and cross-sectional study. We will also exclud studies without sufficient data if additional information cannot be obtained since contacted leading author. About duplicate records, we will exclude 1 which was presented previously or have insufficient information. Any disagreements about the selection of studies will be solved by consulting a third senior reviewer.

### 2.3 Data extraction

Two independent reviewers will utilize the data extraction sheet to perform data extraction. According to the purpose of the present meta-analysis, we will extract the following data: characteristics of studies including the first author name, publication year, and country, information of patients including sample size, age, gender, and body mass index (BMI), details of interventions including regimes of all arms, duration of treatment, measurement tool for outcome, and outcomes of interesting, as well as details of risk of bias of each study. The basic characteristics of all eligible studies will be summarized in Table 2. The corresponding author will be contacted when sufficient information can be not available. Any disagreements about data extraction will be resolved by consulting a third senior investigator.

**Table 2:** Basic characteristics of all eligible studies.

					Details of treatments				
Study	Country	Sample size	Age, yrs	BMI (kg/m2)	Single	Double	Triple	Treatment Duration, mo	Outcomes
Study 1									
Study 2									
Study 3									
Study 4									
Study 5									
Study 6									
Study 7									

### 2.4 Assessment of risk of bias

We will grade the quality of each eligible study according to the assessment result of the risk of bias, which will be appraised with the Cochrane risk of bias assessment tool.^[33]^ Risk of bias will be examined according to the following 6 domains^[34]^: randomization, allocation, blind, incomplete data, selectively reported and other bias sources. A study will be allocated to have a label of “low”, “unclear”, or “high” risk of bias according to the match level between actual information and criteria. Any divergency at risk of bias will be resolved by a third senior investigator. Eventually, we will obtain the overall quality of an individual study (low, moderate or high quality) according to the result of labelling of the risk of bias.

### 2.5 Statistical analysis

In our meta-analysis, all outcomes are measured as a numerical scale with various measurement tools. Therefore, we will express it as standard mean difference (SMD) with 95% confidence interval (CI). We will firstly qualitatively inspect the heterogeneity across studies with the Chinese square test (Q statistic), and then quantitatively estimate the heterogeneity using *I*^*2*^ statistic.^[35]^ All studies will be thought to be homogenesis when *P* > .10 and *I*^*2*^ < 50%. Otherwise, studies will be considered heterogeneous when *P* < .1 and *I*^*2*^ ≥ 50. For studies reporting the standard error of the mean (se), we will calculate the standard deviation (sd) according to the method recommended by Cochrane handbook.^[34]^ When not able to obtain the SD of a record after trying to contact the study authors, we will use the range-rule-of-thumb method to estimate the missing SD.^[36]^ Moreover, we will use net changes in measurements, which will be estimated using the method recommended by Cochrane handbook^[34]^ to estimate the efficacy of all treatments. A *P* value <.05 will be defined as the threshold for statistical significance. We will perform traditional direct meta-analysis based on the random effects model depending on the Review Manager (RevMan) version 5.3 (Cochrane Collaboration, Copenhagen, Denmark). We will drew funnel plot to inspect the possibility of presence of publication bias when the accumulated number of eligible studies for individual outcome was greater than 10.^[37]^

After investigating direct comparisons of single vs multiple injections, single vs double injections, and single vs triple injections, we will also perform network meta-analysis to investigate the comparative efficacy of double and triple injections with Open BUGS software version 3.2.3 (MRC Biostatistics Unit, Cambridge, UK) following methods described by Lu and Ades.^[38,39]^ We will use the initial value which is automatically generated from software to fit the model.^[40]^ To gain convergence, we will perform each Markov chain Monte Carlo chain with 50,000 iterations and 20,000 burn-in. The summary treatment effect estimates will be described as SMD, with 95% credible interval (CrI), for treatment comparisons.

## 3 Discussion

### 3.1 Rational basis of designing this updated network meta-analysis

Knee joint osteoarthritis (KOA) is the main cause of joint degeneration in older individuals. The disease process is characterized by articular cartilage degeneration and whole joint disease.^[3,4]^ These lesions result in joint dysfunction and pain, which can seriously negatively impact the patients quality of life. Intra-articular platelet-rich plasma (PRP) have been extensively utilized to treat KOA, and several meta-analyses have been demonstrated the efficacy of PRP for improving clinical outcomes among KOA patients compared to other intra-articular treatments.^[20,41–43]^ Meanwhile, 2 meta-analyses^[1,30]^ also performed in order to know the appropriate number of injections required to achieve clinical improvement, however conflicting findings were obtained. Moreover, recent studies^[23,28]^ were not included in previous meta-analyses and comparative efficacy of separate multiple injections was not investigated. Therefore, we designed the present meta-analysis to comprehensively address these problems.

### 3.2 Potential mechanisms of PRP for KOA

PRP is a novel growth factor produced via centrifuging autologous whole-blood and isolation of high platelet count plasma.^[44,45]^ Platelets have promising regenerative capacity because their a-granules contain many growth factors such as transforming growth factor-beta, platelet-derived growth factor, epidermal growth factor, vascular endothelial growth factor, fibroblast growth factor, and insulin-like growth factor.^[46,47]^ Moreover, many cytokines (such as unclear factor-kappa B, interleukin-1, and nitric oxide) contained in PRP have also the ability of inhibiting inflammatory effects on chondrocytes.^[48,49]^ Up to now, PRP has been extensively utilized to treat the conditions occurred in bone, cartilage, and soft tissue.^[50]^ Considering the value in promoting regeneration, PRP has also now increasingly been extended to utilize in orthopedics and sport medicine.^[51]^ Certainly, several studies have further demonstrated the possible chondroprotective activity of PRP.^[17]^

### 3.3 Limitations of the present meta-analysis

Regardless of the fact that this current updated network meta-analysis have several advantages compared to previous published meta-analyses, some limitations existed in this study should be cautiously explained. Firstly, we will only search PubMed, Embase and CKNI to identify additional studies, potentially eligible studies indexed in other databases such as SCOPUS may be missed. Second, a published care report^[52]^ have suggested that multiple injections may have positive effective for advanced grades of KOA, however we will not design subgroup analysis to explore the impact of severity of KOA on treatment effects due to limited data after initially screening literatures.

### 3.4 Study status

This updated network meta-analysis has been registered on the INPLASY on November 10, 2020 (Registration Number: INPLASY2020110043). Under the guidance of the PRISMA, our current study has begun, and the search strings have been constructed. We planned to complete data extraction on March 31, 2020 and the report the overall review on December 30, 2020.

### 3.5 Ethics and dissemination

Ethics approval and patients informed consent will not be required in the current updated network meta-analysis because all statistical analyses will be performed based on published data. After completing this updated network meta-analysis, we will communicate our findings through submiting it to peer-reviewed journal and academic conferences.

## Acknowledgments

We would like to deeply appreciate the International Plateform of Registered Systematic Review and Meta-Analysis Protocols (INPLASY) platform for acceptance of our application of registering this protocol.

## Author contributions

PW, KL, and ZJL conceived and designed this protocol. ZXJ, BMQ, CN, and HSL reviewed scoping searches and contributed to the methodological development of the protocol. PW and KL drafted the manuscript and other authors critically made a revision. All authors reviewed and approved the final version for publication. PW is the review guarantor.

**Conceptualization:** Pan Wang, Kai Li, Zhengjiang Li.

**Data curation:** Beiming Qiu, Cheng Nie.

**Formal analysis:** Pan Wang, Kai Li, Zhengjiang Li.

**Investigation:** Zuxin Jiang, Beiming Qiu, Hongsheng Luo.

**Methodology:** Kai Li.

**Resources:** Zuxin Jiang, Beiming Qiu, Cheng Nie, Hongsheng Luo.

**Software:** Pan Wang, Cheng Nie, Hongsheng Luo.

**Supervision:** Zhengjiang Li.

**Writing – original draft:** Pan Wang, Kai Li.

**Writing – review & editing:** Zhengjiang Li.
